# A Rare Case of Salter-Harris Type I Growth Plate Injury in a Patient Undergoing Limb-Lengthening Procedure

**DOI:** 10.7759/cureus.43448

**Published:** 2023-08-14

**Authors:** Syed Mohd Esmat, Ahmad Fadzli Sulong, Mohd Shukrimi Awang, Zhi Sing Oon, Nazri Mohd Yusof

**Affiliations:** 1 Orthopaedics and Traumatology, Sultan Ahmad Shah Medical Centre @International Islamic University Malaysia (IIUM), Kuantan, MYS

**Keywords:** femoral hypoplasia, limb lengthening, limb lengthening procedures, salter-harris injury, growth plate injury

## Abstract

Growth plate injuries over the distal femur typically occur due to high-energy trauma. It is commonly associated with serious complications such as growth disturbance. Its occurrence in children undergoing limb-lengthening procedures is uncommon. We report a case of distal femur growth plate injury in a 13-year-old boy undergoing a limb-lengthening procedure for femoral hypoplasia. Conservative treatment yielded a good functional outcome in this patient.

## Introduction

Type I Salter-Harris (SH) fracture is comparatively rare, especially over the distal femur [1-3]. It is manifested by extra-articular transverse fracture occurring across the growth plate [4]. Most cases are associated with trauma and present a diagnostic challenge as the radiographic evidence of the fracture can often be minimal [4]. Additionally, there are very limited reports on this fracture caused by limb-lengthening procedures. We present a case of a type I SH fracture over the distal femur in a boy with proximal femur focal deficiency undergoing a limb lengthening procedure.

## Case presentation

**Figure 1:**
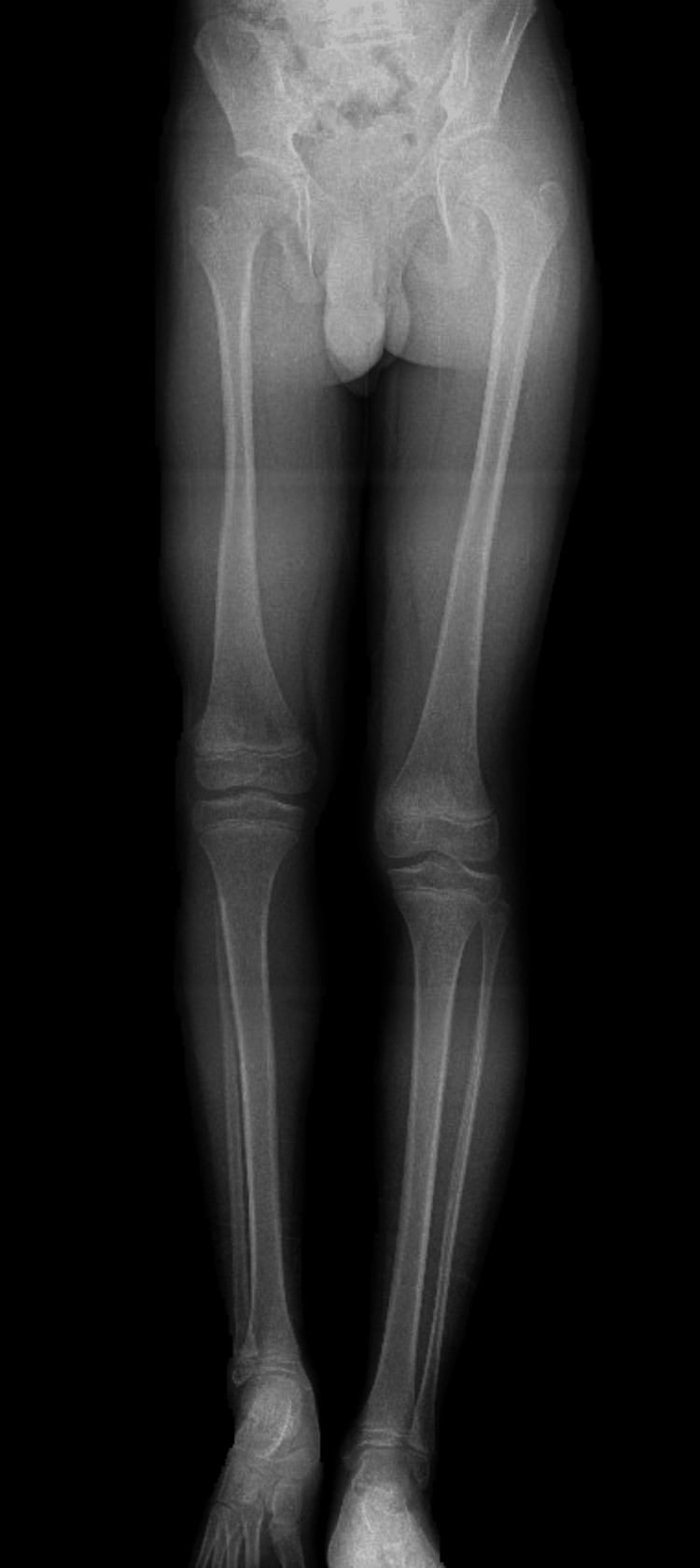
Long leg radiograph revealing right femoral hypoplasia with the absence of fibular head

A 13-year-old boy presented with a painless limp that was becoming more obvious over a period of two years. His physical and motor development was up to age with unremarkable perinatal history. He went to a normal school and was active in sports. Upon examination, he walked unassisted with a short limb gait and there was a 5 cm shortening over the right lower limb. A long leg radiograph revealed a shortened right femur with the absence of the right head of the fibula (Figure 1).

**Figure 2:**
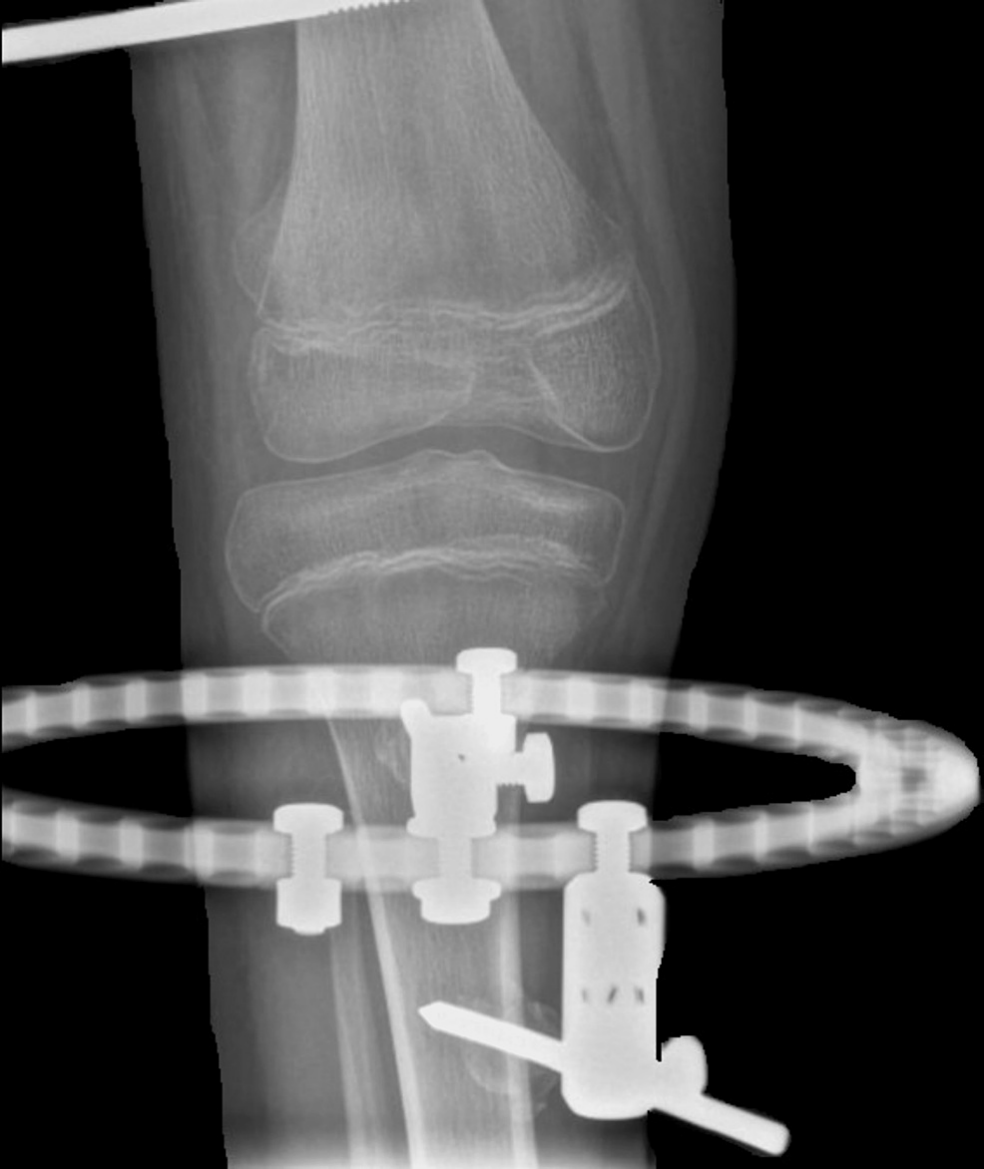
A cross-knee ring external fixator connected to the limb reconstruction system

He was diagnosed with right proximal femur focal deficiency and underwent a limb-lengthening procedure with a hybrid limb reconstruction system (LRS). A cross-knee ring external fixator was constructed and connected to the LRS to prevent knee subluxation during lengthening (Figure 2). Intraoperatively, great precautions were taken to avoid any direct injury to the growth plate. The operation was uneventful, and the lengthening phase commenced one week after the index procedure. The duration of the lengthening phase was 50 days, with a rate of 1 mm lengthening per day. The duration of consolidation was about three months after the end of the lengthening phase. Additionally, the osteotomy site was located 10 cm proximal to the distal femoral growth plate. In total, 5 cm of femoral bone lengthening was achieved. The cross-knee ring external fixator was removed from the LRS after the completion of the lengthening phase. The patient started having pain over the right knee after the removal of the cross-knee ring external fixator which worsened upon passive movement. He denied any history of recent falls or trauma prior to the knee pain.

**Figure 3:**
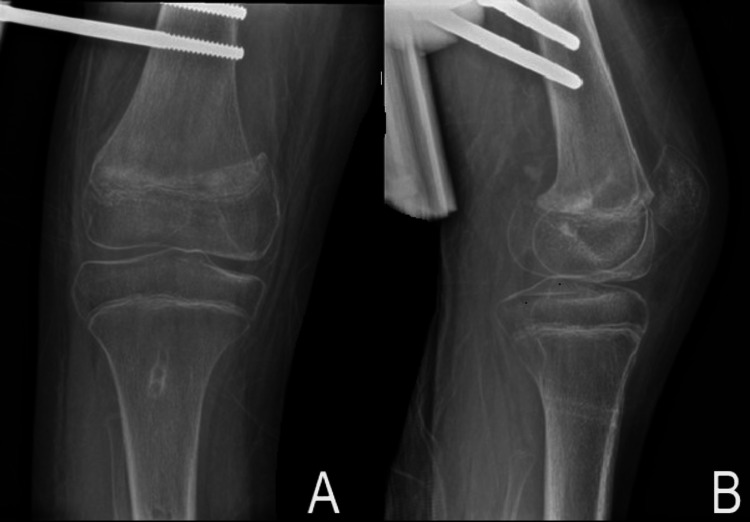
Radiographs of the right knee Anteroposterior (AP) (A) and lateral (B) radiograph of the right knee displaying type I Salter-Harris fracture over the distal femur.

Physical examination of the knee revealed slight swelling with tenderness over the medial condyle of the femur. A plain radiograph of the knee displayed a 2 mm medial separation of the distal femoral growth plate with no fracture extension to the metaphysis or epiphysis (Figure 3). On the lateral view, there was no anterior or posterior displacement over the physis.

**Figure 4:**
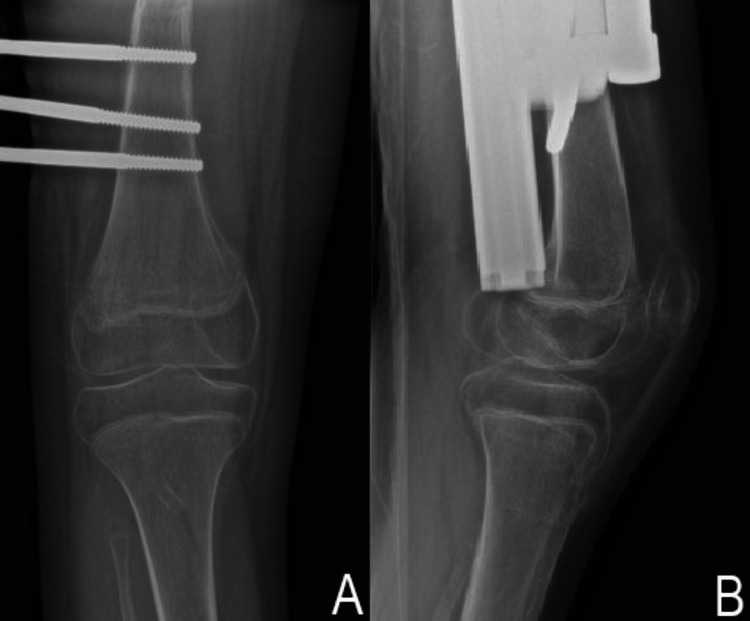
Repeat radiographs of the right knee Anteroposterior (AP) (A) and lateral (B) radiographs of the right knee showing callus formation surrounding the fracture site.

He was diagnosed with type I SH fracture of the distal femur and treated conservatively. Immobilization for six weeks followed by gentle knee range of motion exercise was accomplished. After six months, he had a full knee range of motion and was able to ambulate with aid. A repeated plain radiograph showed a progressive callus formation surrounding the growth plate with no further displacement of the fracture (Figure 4).

## Discussion

In a growing bone, the transitional zone between the growth plate and the bone is considered the weakest section vulnerable to injury. This is aggravated throughout puberty as the growth plate thickens during a rapid growth spurt as experienced by our patient [5]. Salter and Harris originally classified growth plate injuries based on the anatomical extension of the fractures and their prognosis [6]. According to them, type I fracture occurs when there is a direct injury of the growth plate resulting in a separation between the epiphysis and metaphysis. During the bone lengthening process, the gradual stretch of the surrounding muscles may lead to plastic deformation and subsequent contractures. When bone is lengthened by more than 30% of its initial length, there are visible histological changes in the surrounding muscular structures [7]. The direct effect of bone lengthening on the growth plates has not been observed clinically as reported by Hope et al. [8]. In our case, we believe the growth plate injury occurred indirectly due to the compression caused by the tight surrounding musculatures following the removal of the cross-knee external fixator. The fracture may also be precipitated by the poor condition of the bone due to disuse osteoporosis. The direct effect of the growth plate injury from the lengthening process was unlikely as the total lengthening was only 16% of the overall length of the femur.

Fracture displacement occurs frequently in the distal physis of the femur, especially in the coronal plane. In general, anatomical reduction with surgical fixation is advocated in displaced fractures while conservative treatment is recommended in minimally displaced fractures. In a retrospective study of 83 distal femur growth plate fractures, more than 60% of the fractures were classified as minimally displaced [1]. The authors defined minimally displaced fractures as those displaced less than 1/3 of the bone width. In their series, displaced type I SH fractures were also treated conservatively per the surgeon’s preference. They reported a lower complication rate in the group treated conservatively but recognized the possibility of selection bias whereby surgical treatment was preferred in more severe fractures. Another retrospective study of 151 patients with distal femoral physis fractures revealed a high rate of reduction loss in patients treated with long leg casts [3]. In the current case, the fracture was minimally displaced, and no loss of reduction was observed on serial radiographs.

The distal femoral growth plate provides 70% of femoral length with the fastest growing rate of 10 mm per year [4,8]. Injury to the growth plate at this level was associated with a high rate of complications of up to 50% [9]. Most authors reported growth disturbance as the most common complication and may occur in the form of malalignment or limb length discrepancy [1,3]. When compared to other subtypes of SH fractures, type I fractures were associated with the lowest risk of growth disturbances in a meta-analysis of 564 fractures [2]. Bellamy et al. concluded that the severity of fracture displacement and remaining years of growth until skeletal maturity was associated with an increased risk of requiring corrective surgery for growth disturbance [10]. In addition to fracture displacement, Arkader et al. stated that the severity of fracture according to SH classification was significantly correlated with the incidence of growth disturbance [1]. The majority of the patients required excision of physeal bar or contralateral epiphysiodesis to manage the limb length discrepancy.

## Conclusions

The occurrence of SH type I growth plate injury in patients undergoing limb-lengthening procedures is rare. Excessive bone lengthening must be avoided to prevent injuries to the surrounding soft tissue structures including the growth plate. Any risk of growth plate injury after the removal of the frame should be scrutinized. Dire consequences of growth disturbance may result if it is not timely recognized and treated accordingly. Meticulous clinical assessment with serial radiographs plays a vital role in conservative treatment to ensure that there was no loss of reduction that may lead to malignment or limb length discrepancy. A longer duration of follow-up in the current case is paramount in detecting the possibility of growth disturbance. Finally, SH type I growth plate injury is a stable injury and conservative treatment provides good clinical outcomes.
